# Involvement of conformational isomerism in the complexity of the crystal network of 1-(4-nitro­phen­yl)-1*H*-1,3-benzimidazole derivatives driven by C—H⋯*A* (*A* = NO_2_, N_py_ and π) and orthogonal N_py_⋯NO_2_ and ONO⋯C*sp* ^2^ inter­actions

**DOI:** 10.1107/S2053229618003406

**Published:** 2018-03-07

**Authors:** Mónica I. García-Aranda, Carlos Z. Gómez-Castro, Efrén V. García-Báez, Yolanda Gómez y Gómez, José L. Castrejón-Flores, Itzia I. Padilla-Martínez

**Affiliations:** a Laboratorio de Química Supramolecular y Nanociencias, Unidad Profesional Interdisciplinaria de Biotecnología del Instituto Politécnico Nacional, Av. Acueducto s/n Barrio la Laguna Ticomán, 07340 Mexico City, Mexico; b Laboratorio de Farmacología, Unidad Profesional Interdisciplinaria de Biotecnología del Instituto Politécnico Nacional, Av. Acueducto s/n Barrio la Laguna Ticomán, 07340 Mexico City, Mexico; c Laboratorio de Biotecnología Molecular, Unidad Profesional Interdisciplinaria de Biotecnología del Instituto Politécnico Nacional, Av. Acueducto s/n Barrio la Laguna Ticomán, 07340 Mexico City, Mexico

**Keywords:** orthogonal nitro⋯C inter­action, crystal structure, orthogonal nitro⋯N inter­action, helix, conformational isomerism, high-*Z*′ structure, snapshot conformer

## Abstract

A detailed structural analysis of the benzimidazole nitro­arenes 1-(4-nitro­phen­yl)-1*H*-1,3-benzimidazole, C_13_H_9_N_3_O_2_, (I), 1-(4-nitro­phen­yl)-2-phenyl-1*H*-1,3-benzimidazole, C_19_H_13_N_3_O_2_, (II), and 2-(3-methyl­phen­yl)-1-(4-nitro­phen­yl)-1*H*-1,3-benzimidazole, C_20_H_15_N_3_O_2_, (III), has been performed. They are nonplanar structures whose crystal arrangement is governed by C*sp* ^2^—H⋯*A* (*A* = NO_2_, N_py_ and π) hydrogen bonding. The inherent complexity of the supra­molecular arrangements of compounds (I) (*Z*′ = 2) and (II) (*Z*′ = 4) into tapes, helices and sheets is the result of the additional participation of π– and *n*–π* (*n* = O and N_py_; π* = C*sp* ^2^ and ) inter­actions that contribute to the stabilization of the equi-energetic conformations adopted by each of the independent mol­ecules in the asymmetric unit. In contrast, compound (III) (*Z*′ = 1) is self-paired, probably due to the effect of the steric demand of the methyl group on the crystal packing. Theoretical *ab initio* calculations confirmed that the presence of the arene ring at the benzimidazole 2-position increases the rotational barrier of the nitro­benzene ring and also supports the electrostatic nature of the orthogonal ONO⋯C*sp* ^2^ and N_py_⋯NO_2_ inter­actions.

## Abstract 2

Participation of π–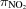 and *n*–π* (*n* = O and N_py_; π* = C*sp* ^2^ and 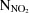) inter­actions in the equi-energetic conformations of 1-(4-nitro­phen­yl)-1*H*-1,3-benzimidazoles.

## Introduction   

Benzimidazoles are recognized as essential chemical motifs present in a variety of natural products, agrochemicals and bioactive mol­ecules (Keri *et al.*, 2015 ▸). Particularly, C2-aryl-substituted benzimidazoles are often found as a key unit in various natural compounds, biologically active agents, potent pharmacophores and functional chemicals (Horton *et al.*, 2003 ▸; Kumar, 2004 ▸; Candeias *et al.*, 2009 ▸; Gupta & Rawat, 2010 ▸; Carvalho *et al.*, 2011 ▸). In addition, *N*-aryl­benzimidazoles are a class of prominent heterocyclic compounds that exhibit a wide range of biological properties (Sabat *et al.*, 2006 ▸; Elias *et al.*, 2011 ▸). In particular, 1,2-di­aryl­benzimidazoles have been reported as strong inhibitors of human cyclo­oxygenases with a skewed selectivity towards the COX-2 (cyclooxygenase 2) isoform at the micromolar level (Secci *et al.*, 2012 ▸).

It is worth mentioning the case of 1-(4-nitro­phen­yl)-1*H*-1,3-benzimidazole, (I), which has been reported as an inhibitor of platelet-derived growth factor receptor (PDGFR), which is highly expressed in tumour cells (Zhong *et al.*, 2004 ▸; Katritzky *et al.*, 2005 ▸). Experimental evidence indicates that the inhibitory activity involves discrete noncovalent dipolar protein–ligand inter­actions, which significantly contribute to the binding affinity and to inter­molecular recognition. On the other hand, little is known about the nature of the noncovalent inter­actions of nitro­arenes with hydro­phobic aromatic protein 
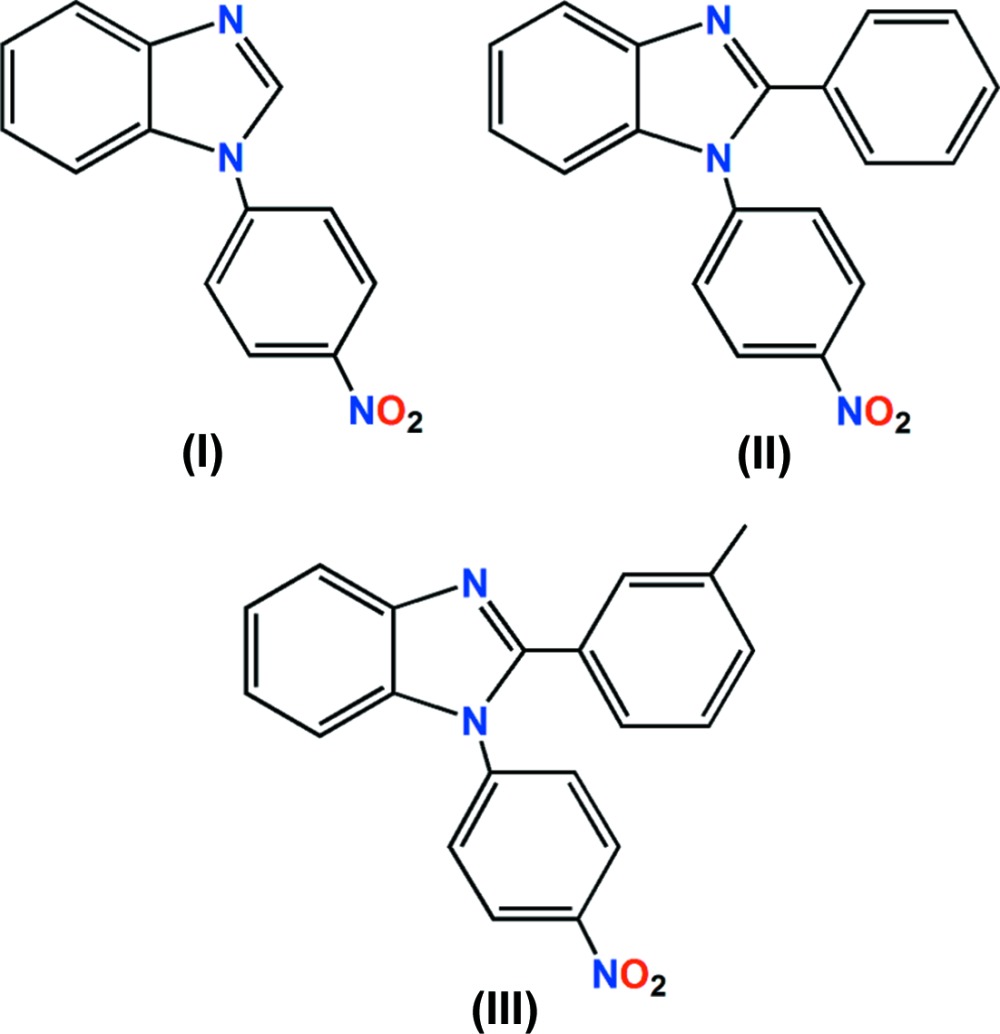
areas and their contribution to binding affinities, which might be relevant for the inter­action with different receptors (An *et al.*, 2015 ▸). In this context, 1-(4-nitro­phen­yl)-2-phenyl-1*H*-1,3-benzimidazole, (II), and 2-(4-methyl­phen­yl)-1-(4-nitro­phen­yl)-1*H*-1,3-benzimidazole, (III), were also synthesized and their mol­ecular structures analysed with the aim of further understanding their pharmacophore properties, as well their use in the design of materials with specific functions. Moreover, compounds (I)–(III) are characterized by the presence of strong hydrogen-bond acceptor groups but weak hydrogen-bond donors, allowing us to further expand our knowledge of the roles of noncovalent inter­molecular forces in crystal engineering and supra­molecular chemistry.

## Experimental   

### Instrumental   

The uncorrected melting points were measured in open-ended capillary tubes in an Electrothermal apparatus IA 9100. ^1^H (300.01 MHz) and ^13^C NMR (75.46 MHz) spectra were recorded on a Varian Mercury-300 spectrometer using CDCl_3_ as solvent and tetra­methyl­silane (TMS) as inter­nal reference; chemical shift values (δ) are in parts per million (ppm) and coupling constants (*J* values) are in Hertz (Hz). IR spectra was obtained with a 3100 FT–IR Excalibur Series spectrophotometer.

### Theoretical calculations   

Geometry optimizations at the B3LYP/6-31G(d,p) level of theory were performed without any symmetry restraints using the *GAUSSIAN09* package (Frisch *et al.*, 2009 ▸). Relaxed linear potential energy surface scans for the N1—C10 and C2—C16 rotations were performed using direct inversion of iterative subspace (GDIIS) (Farkas & Schlegel, 2002 ▸).

### Synthesis and crystallization   

#### 1-(4-Nitro­phen­yl)-1*H*-1,3-benzimidazole, (I)   

Com­pound (I) was prepared from benzimidazole (1.00 g, 8.47 mmol) and 1-fluoro-4-nitro­benzene (1.19 g, 8.47 mol) in a basic medium of K_2_CO_3_ (2.34 g, 16.9 mmol) in dimethyl sul­foxide (13 ml) at 393 K for 20 h, as reported for 2-(4-bromophenyl)-1-(4-nitrophenyl)-1*H*-benzimidazole (González-Padilla *et al.*, 2014 ▸). The com­pound was obtained as a pale-yellow solid in 96% yield (m.p. 453–454 K). Crystals of (I) were obtained after crystallization from an ethanol solution. ^1^H NMR: δ 8.48 (*m*, 2H, H-12,14), 8.20 (*s*, 1H, H-2), 7.92 (*m*, 1H, H-4), 7.41 (*m*, 2H, H-5,6), 7.62 (*m*, 1H, H-7), 7.77 (*m*, 2H, H-11,15). ^13^C NMR: δ 146.7 (C-13), 144.6 (C-9), 141.9 (C-10), 141.8 (C-2), 133.0 (C-8), 126.1 (C-12,14), 124.8 (C-6), 124.0 (C5), 123.9 (C-11,15), 121.4 (C-4), 110.5 (C-7). IR (neat, ν, cm^1^): 1595, 1507 (C=C Ar), 1347 (NO_2_), 848, 754 (C—H out of plane).

#### 1-(4-Nitro­phen­yl)-2-phenyl-1*H*-1,3-benzimidazole, (II)   

Compound (II) was prepared from 2-phenyl-1*H*-1,3-benzimidazole (0.216 g, 1.11 mmol) and 1-fluoro-4-nitro­ben­zene (0.157 g, 1.11 mmol) in a basic medium of K_2_CO_3_ (0.155 g, 1.11 mmol), di­methyl­formamide (2 ml) and CuCl (11 mg) as catalyst, as a yellow solid in 72% yield (m.p. 421–423 K). Crystals suitable for X-ray diffraction were obtained from a hexa­ne/ethyl acetate solution. ^1^H NMR: δ 8.34 (*m*, 2H, H-12,14), 7.88 (*d*, 1H, H-4, *^3^J* = 7.6 Hz), 7.48 (*m*, 4H, H-11,15,17,21), 7.34 (*m*, 6H, H5-7, 18-20). ^13^C NMR: δ 152.4 (C2), 147.2 (C9), 143.4 (C13), 142.8 (C10), 136.4 (C8), 130.2 (C19), 129.7 (C16), 129.5 (C18,20), 128.9 (C17), 128.1 (C12,14), 125.5 (C11,15), 124.3 (C6), 124.0 (C5), 120.6 (C4), 110.1 (C7). IR (neat, ν, cm^1^): 1590, 1526 (C=C Ar), 1350 (NO_2_), 778, 746, 694 (C—H out of plane).

#### 2-(4-Methyl­phen­yl)-1-(4-nitro­phen­yl)-1*H*-1,3-benz­imidazole, (III)   

Compound (III) was prepared from 2-*m*-tolyl-1*H*-1,3-benzimidazole (0.300 mg, 1.44 mmol) as a yellow solid in 20% yield after silica-gel chromatography (m.p. 447.7–449.0 K). Crystals suitable for X-ray diffraction were obtained from an hexa­ne/ethyl acetate solution. ^1^H NMR: δ 8.38 (*m*, 2H, H-18,20), 7.90 (*d*, 1H, H-4, ^3^ *J* = 7.8 Hz), 7.54 (*s*, 1H, H15), 7.50 (*m*, 2H, H-17,21), 7.34 (*m*, 6H, H5-7, 11-13), 2.34 (*s*, 3H, Me). ^13^C NMR: δ 152.7 (C2), 147.2 (C9), 143.4 (C10), 142.8 (C13), 139.0 (C20), 136.3 (C8), 131.1 (C18), 130.5 (C17), 129.3 (C16), 128.6 (C19), 128.1 (C12,14), 126.7 (C21), 125.1 (C11,15), 124.2 (C6), 124.0 (C5), 120.6 (C4), 110.1 (C7), 21.6 (Me). IR (neat, ν, cm^1^): 1678 (C=N), 1590, 1515 (C=C Ar), 1347, 1303 (NO_2_), 791, 756, 745, 695 (C—H out of plane).

### Refinement   

Crystal data, data collection and structure refinement details are summarized in Table 1 ▸. H atoms on C atoms were positioned geometrically and treated as riding atoms, with C—H = 0.95–0.99 Å and *U*_iso_(H) = 1.5*U*_eq_(C) for methyl H atoms or 1.2*U*_eq_(C) otherwise.

## Results and discussion   

### Mol­ecular and supra­molecular structure of compound (I)   

**Figure 1:**
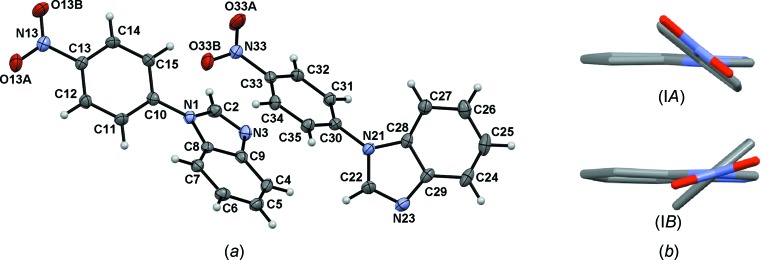
(*a*) The mol­ecular structure of compound (I), with displacement ellipsoids drawn at the 30% probability level. Two independent mol­ecules, *i.e.* (I*A*) (atoms N1/C2/N3/C4–C15/N13/O13*A*/O13*B*) and (I*B*) (N21/C22/N23/C24-C35/N33/O33*A*/O33*B*), are present in the asymmetric unit. (*b*) A view of rotamers (I*A*) and (I*B*) along the N13⋯N3 and N33⋯N23 imaginary axes, respectively.

Two independent mol­ecules, *i.e. A* (atoms N1/C2/N3/C4–C15/N13/O13*A*/O13*B*) and *B* (N21/C22/N23/C24–C35/N33/O33*A*/O33*B*), appear in the asymmetric unit of compound (I) (Fig. 1 ▸), which crystallizes in the monoclinic space group *C*2/*c*. Mol­ecules (I*A*) and (I*B*) are related by a second-order pseudo-helicoidal axis. The nitro­benzene ring (denoted N-nitroBz) is twisted from the mean benzimidazole (Bzm) plane by 35.71 (9) and 40.11 (7)° in mol­ecules (I*A*) and (I*B*), respectively (Spek, 2009 ▸). The first value is very close to that observed in 6-meth­oxy-1-(4-nitro­phen­yl)-1*H*-1,3-benzimidazole (36.15°; Kumar *et al.*, 2013 ▸). The NO_2_ group is in the plane of the nitro­benzene ring in (I*A*) [C14—C13—N13—O13*B* = −2.2 (4)°] and twisted in (I*B*) [C34—C33—N33—O33*B* = −22.4 (4)°]. However, the C—NO_2_ bond lengths are equal in both mol­ecules and are also in the expected range (Allen *et al.*, 1987 ▸), suggesting a limited conjugation between them. The N-nitroBz ring in the reported crystal structures of 1-(4-nitro­phen­yl)pyrazole and 1-(4-nitro­phen­yl)pyrrole (Ishihara *et al.*, 1992 ▸) is almost coplanar with the heterocyclic ring. Thus, the observed twist of the N-nitroBz ring from the Bzm plane in compound (I) is the result of steric repulsion between the fused benzene and N-nitroBz rings. This last ring can adopt a perpendicular disposition, in relation to the Bzm heterocycle, similar to those structures with high steric demand such as phenanthro­imidazoles (Zhang *et al.*, 2016 ▸).

**Figure 2:**
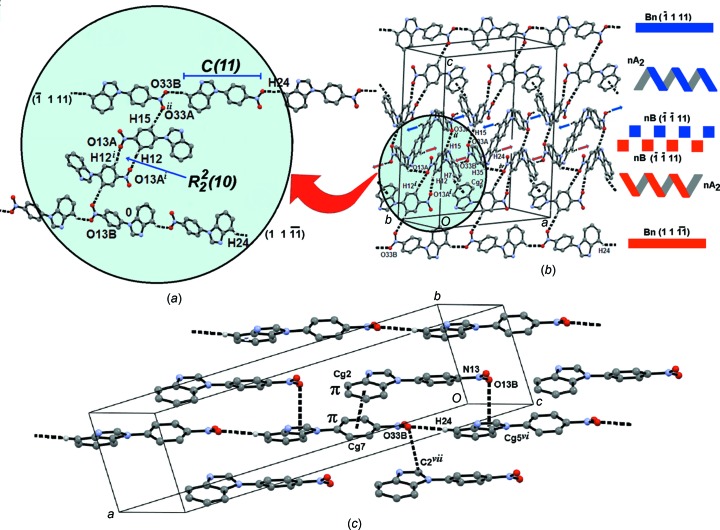
The two-dimensional supra­molecular architecture of compound (I), built up by C—H⋯O inter­actions. (*a*) Dimers *A*_2_ and tapes *B_n_* are shown. (*b*) The *M* and *P* strands formed by the inter­linkage of tapes, and helices *B_n_nA*_2_ *nB*. (*c*) Dispersive inter­actions ONO⋯*Cg*, *Cg*⋯*Cg* and ONO⋯C2, giving rise to the three-dimensional network of compound (I).

Soft C*sp* ^2^—H⋯O inter­actions give shape to the crystal packing, with the participation of a nitro O atom, as the acceptor, in a monocoordination fashion (Allen *et al.*, 1997 ▸). Two (I*A*) mol­ecules form centrosymmetric dimers, *i.e.* *A*_2_, through C12—H12⋯O13*A* ^i^ inter­actions, describing a twisted 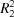
(10) motif (Bernstein *et al.*, 1995 ▸) (Fig. 2 ▸ *a*). Furthermore, a *meso* helix is developed along the [030] direction through C7—H7⋯*Cg*2^v^ T-shaped inter­actions linking the *A*_2_ dimers [*Cg*2 is the centroid of the C4–C9 ring; symmetry code: (v) −*x* + 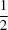, *y* − 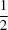, −*z* + 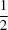]. (I*B*) mol­ecules self-associate into *C*(11) chains through C24—H24⋯O33*B* ^iii^ inter­actions, which propagate within the (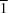,1,11) and (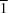,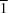,11) families of planes. *A* and *B* mol­ecules of compound (I) are connected through C15—H15⋯O33*A* ^ii^ inter­actions. Chains of (I*B*) running in the [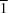,1,11] direction and *n* mol­ecules of (I*B*), each belonging to an infinite number of (I*B*) chains running within the (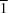,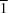,11) family of planes, are linked to *A*_2_ helices, forming the *M* and *P* strands *B_n_nA*_2_ *nB*. The second dimension is given by the inter­linkage of the strands through C35—H35⋯O13*A* ^iv^ inter­actions (Figs. 2 ▸ *b* and 2*c*). The geo­metric features and symmetry codes associated with these inter­actions are listed in Table 2 ▸. Mol­ecule (I*B*) displays a twist of 22.4 (4)° of the NO_2_ group, which is comparable to that seen in high-energy mol­ecules such as TNT (Landenberger & Matzger, 2010 ▸). This torsion, together with an N-nitroBz torsion of 40.11 (7)° from the Bzm plane, favour the helical arrangement of (I) in the solid (Ramírez-Milanés *et al.*, 2017 ▸).

The three-dimensional structure is developed by nitro–π and π–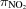 dispersive inter­actions, *viz.* the nitro group of mol­ecule (I*A*) to the centroid of the heterocyclic ring of mol­ecule (I*B*), *i.e.* N13⋯O13*B*⋯*Cg*5^vi^ [O13*B*⋯*Cg*5 = 3.346 (3) Å, N13⋯*Cg*5 = 3.431 (3) Å and N13⋯O13*B*⋯*Cg*5 = 83.68 (19)°; *Cg*5 is the centroid of the N21/C22/N23/C29/C28 ring; symmetry code: (vi) *x* − 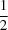, *y* − 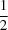, *z*], and *Cg*2⋯*Cg*7^v^, between the aromatic ring (*Cg*2 is the centroid of the C4—C9 ring and *Cg*7 is the centroid of the C30–C35 ring) of the Bzm moiety of mol­ecule (I*A*) and the nitro­benzene ring of mol­ecule (I*B*). The inter­centroid *Cg*2⋯*Cg*7^v^ distance [3.6123 (17) Å] is very close to the inter­planar distance [3.4361 (12) Å], in agreement with a face-to-face inter­action (García-Báez *et al.*, 2003 ▸). It is worth mentioning that the calculated value of the gas-phase binding energy of π–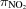 stacking has been reported as −6.7 kcal mol^−1^ (1 kcal mol^−1^ = 4.184 kJ mol^−1^) between phenyl­alanine and nitro­benzene (An *et al.*, 2015 ▸), pointing to the relevance of this inter­action in the crystal lattice arrangement.

In addition, an inter­molecular NO_2_⋯C*sp* ^2^ inter­action is observed between a nitro O atom as donor and the C2 atom of the NCN fragment of the heterocyclic Bzm ring. The geometric parameters associated with this last *n*–π* inter­action are O33*B*⋯C2^vii^ = 3.213 (3) Å and N33⋯O33*B*⋯C2 = 93.7 (2)° [sym­metry code: (vii) *x*, *y* − 1, *z*] (Fig. 2 ▸ *c*). This inter­action has been described in 3,3′-di­nitro-2,2′-bi­pyri­dine *N*-oxides, with distances in the range 2.762 (4)–2.789 (3) Å (O’Leary & Wallis, 2007 ▸), clearly shorter than in (I) because of its intra­molecular nature. Additionally, an analogous inter­action of the nitrile group with the C2 atom of the Bzm ring, CN⋯C*sp* ^2^, occurred in (*Z*)-3-(4-nitro­phen­yl)-2-(1-phenyl-1*H*-benzimidazol-2-yl)acrylo­nitrile (Hranjec *et al.*, 2012 ▸).

### Mol­ecular and supra­molecular structure of compound (II)   

**Figure 3:**
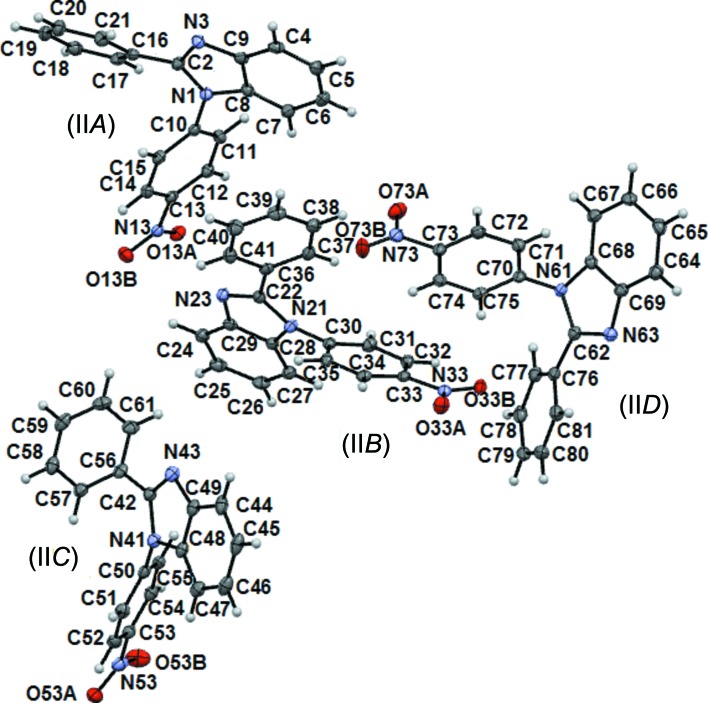
The mol­ecular structure of compound (II), with displacement ellipsoids drawn at the 30% probability level. Four independent mol­ecules are present in the asymmetric unit, namely *A* (atoms N1/C2/N3/C4–C15/N13/O13*A*/O13*B*), *B* (N21/C22/N23/C24–C35/N33/O33*A*/O33*B*), *C* (N41/C42/N43/C44–C55/N53/O53*A*/O53*B*) and *D* (N61/C62/N63/C64–C75/N73/O73*A*/O73*B*).

Compound (II) crystallizes in the triclinic space group *P* 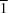, with four independent mol­ecules in the asymmetric unit (Fig. 3 ▸), namely (II*A*) (atoms N1/C2/N3/C4–C15/N13/O13*A*/O13*B*), (II*B*) (N21/C22/N23/C24–C35/N33/O33*A*/O33*B*), (II*C*) (N41/C42/N43/C44–C55/N53/O53*A*/O53*B*) and (II*D*) (N61/C62/N63/C64–C75/N73/O73*A*/O73*B*). Mol­ecules (II*A*) and (II*C*), as well as (II*B*) and (II*D*), are related by a local pseudocentre of inversion located at the fractional coordinates (0.276, 0.376, 0.626) and (0.272, 0.876, 0.626), respectively, in the asymmetric unit. This condition has frequently been observed in *P* 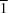 crystals of high-*Z*′ structures (Desiraju, 2007 ▸). The N-nitroBz and C2-Ph rings are both twisted from the mean Bzm plane; the angles between the planes of the Bzm, N-nitroBz and C2-Ph rings are listed in Table 3 ▸. In spite of their inherent crystallographic differences, mol­ecules (II*A*) and (II*B*) have similar angles, as have mol­ecules (II*C*) and (II*D*), judged by the mean values of the angles between the planes. The N-nitroBz ring in compound (II) deviates more from coplanarity with the Bzm ring than the C2-Ph ring, but within the range found for 1,2-di­phenyl­benzimidazole compounds (González-Padilla *et al.*, 2014 ▸) and 1-(4-nitro­phen­yl)-2-phenyl­imidazole (Ishihara *et al.*, 1992 ▸).

**Figure 4:**
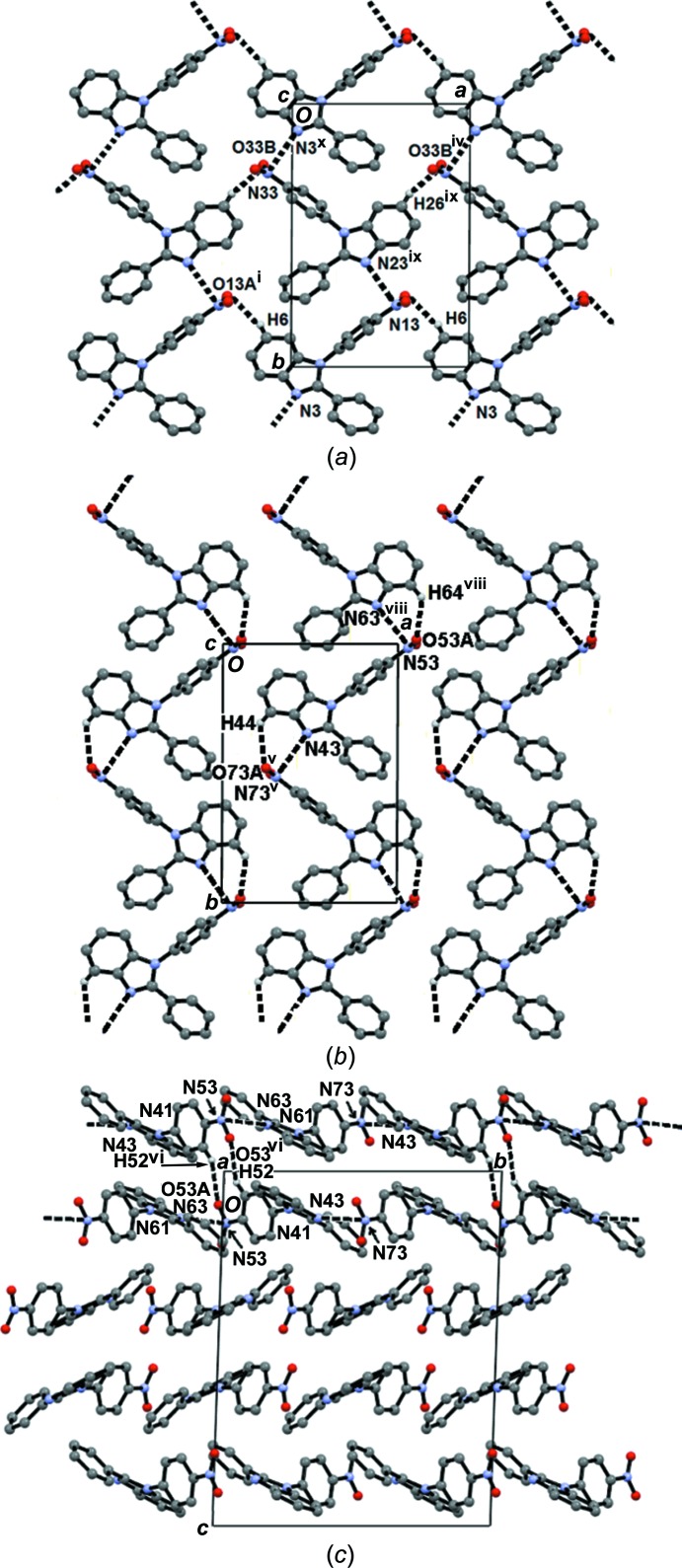
The supra­molecular architecture of compound (II). (*a*) The inter­linkage of 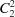
(16) chains propagating along the *b* axis developing the two-dimensional arrangement of mol­ecules (II*A*) and (II*B*) in the *ab* plane. (*b*) Ribbons of (II*C*)/(II*D*) developing along the *b* axis; the (II*D*) mol­ecule with symmetry code (*x*, *y*, *z*) is not shown for clarity. (*c*) (*DC*_2_ *D*)_*n*_ ribbons within the *bc* plane.

Mol­ecules (II*A*) and (II*B*) are linked through N⋯NO_2_ inter­actions (N13⋯N23 and N33⋯N3), with the participation of the pyridine-like N atom as the donor and the N atom of the nitro group as the acceptor, forming 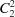
(16) chains propagating along the *b* axis. Chains of (II*A*) and (II*B*) mol­ecules are linked through C6—H6⋯O13*A* ^i^ and C26—H26⋯O33*B* ^iv^ soft hydrogen bonds to develop a sheet within the *ab* plane. C7—H7⋯O33*B* ^ii^ and C27—H27⋯O13*A* ^v^ soft hydrogen bonds are responsible for linking two (II*A*)/(II*B*) planes along the *c*-axis direction, *i.e.* (*A*_2_ *B*_2_)_*n*_ (Fig. 4 ▸ *a*). These C—H⋯O inter­actions are of the bifurcated type with respect to the acceptor O atoms, *i.e.* H6⋯O13*A*⋯H27 and H7⋯O33*B*⋯H26. The geometrical parameters and symmetry codes of the hydrogen-bonding and N⋯NO_2_ inter­actions are listed in Tables 4 ▸ and 5 ▸, respectively.

Mol­ecules (II*C*) and (II*D*) develop a (*DC*_2_ *D*)_*n*_ ribbon within the (10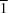) family of planes (Figs. 4 ▸ *b* and 4*c*), also through N⋯NO_2_ and C—H⋯O inter­actions (N63⋯N53^viii^, N43⋯N73^v^, C44—H44⋯O73*A* ^i^, C64—H64⋯O53*A* ^viii^ and C52—H52⋯O53*A* ^vi^). The (*A*_2_ *B*_2_)_*n*_ double sheets and (*DC*_2_ *D*)_*n*_ ribbons are inter­leaved to develop the three-dimensional structure along the *c*-axis direction through C—H⋯*X* (*X* = N and O) inter­actions (C54—H54⋯N3^vii^, C74—H74⋯N23^v^, C15—H15⋯O53*B* ^iii^, C35—H35⋯O73*B* ^v^, C55—H55⋯O33*A* ^iv^ and C75—H75⋯O13*B* ^v^), with the participation of the pyridine-like N and nitro O atoms as acceptors.

Remarkably, the inter­molecular N_py_⋯NO_2_ (*n*–π*) inter­action plays a crucial role in the mol­ecular self-assembly and crystal packing of compound (II). The nitro N atoms have been observed to inter­act with electron-rich centres, such as an O atom of another nitro group (Daszkiewicz, 2013 ▸), the N atom of a di­methyl­amino group in peri­naphthalenes (Egli *et al.*, 1986 ▸; Ciechanowicz-Rutkowska, 1977 ▸) and the pyridine-like N atom of azole compounds (Yap *et al.*, 2005 ▸). The geometric parameters of the inter­molecular N⋯NO_2_ inter­actions in (II) are similar to the values found in the crystal structure of 2-methyl-4,6-di­nitro-1-(2,4,6-tri­nitro­phen­yl)benz­imidazole (Freyer *et al.*, 1992 ▸), with N⋯N = 3.089 Å and C⋯N⋯N = 95.4°.

The NO_2_⋯C*sp* ^2^ and N_py_⋯NO_2_ inter­actions present in (I) and (II), respectively, are of the orthogonal *n*–π* type, since the donor atom approaches in a perpendicular manner to the plane that includes the acceptor. N⋯NO_2_ inter­actions have been envisaged as an entry to supra­molecular cages without using metals to make orthogonal corners (Yap *et al.*, 2005 ▸).

The crystal structure of compound (II) is an example of a compound with many symmetry-independent mol­ecules in the asymmetric unit. This phenomenon has been extensively analysed elsewhere (Bernstein *et al.*, 2008 ▸). The introduction of a C2-Ph ring in compound (II) to the already present N-nitroBz ring in (I) is expected to increase the rotational barrier of the latter, reducing the possibilities of conformational isomers. Nevertheless, the effect is the opposite and contrasts with similar structures lacking the nitro group, such as 1-phenyl-2-*p*-tolyl-1*H*-benzimidazole (Mohandas *et al.*, 2013 ▸) and 1,2-diphenyl-1*H*-benzimidazole (Rosepriya *et al.*, 2012 ▸), or those containing a nitro group, 6-ethyl-1-(4-nitro­phen­yl)-2-phenyl-1*H*-benzimidazole (Kumar & Punniyamurthy, 2012 ▸), but having steric constraints. All of them only have one mol­ecule in the asymmetric unit.

### Mol­ecular and supra­molecular structure of compound (III)   

**Figure 5:**
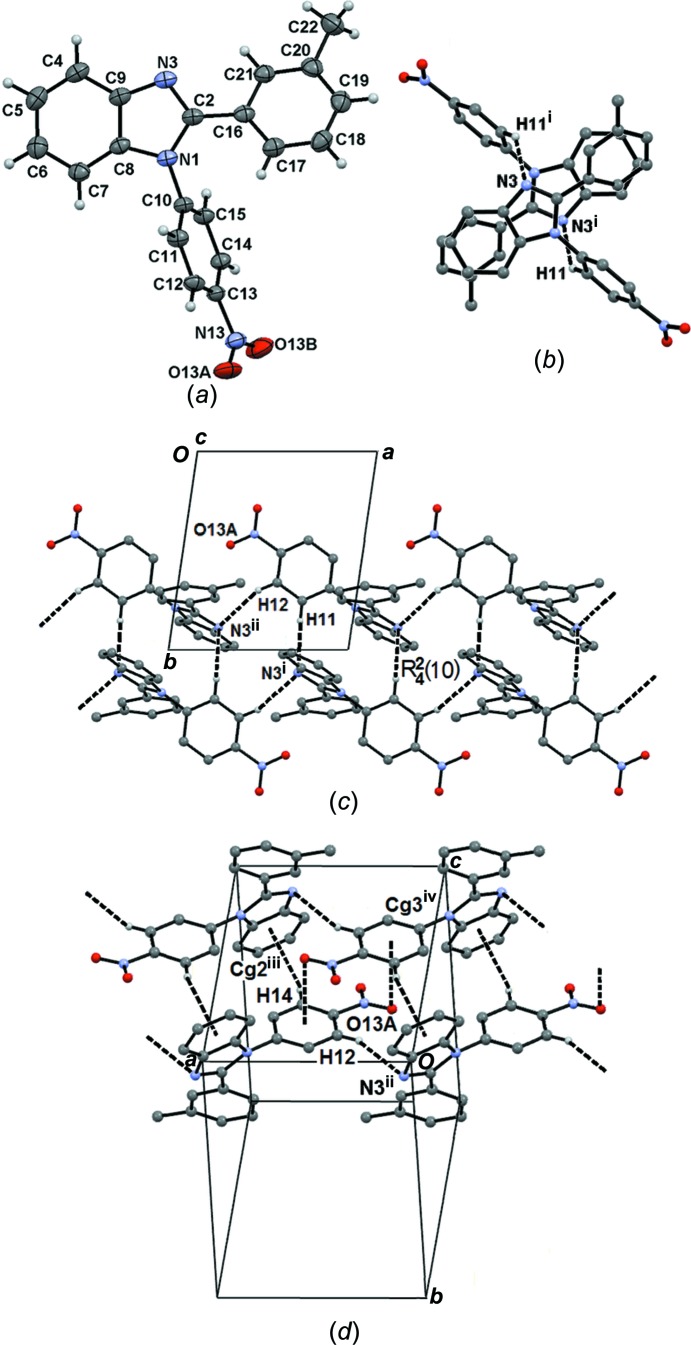
(*a*) The mol­ecular structure of compound (III), with displacement ellipsoids drawn at the 30% probability level. The supra­molecular structure as (*b*) zero-dimensional, (*c*) one-dimensional, and (*d*) two- and three-dimensional.

Compound (III) crystallizes in the triclinic space group *P* 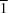, with one mol­ecule in the asymmetric unit (Fig. 5 ▸ *a*). Both N-nitroBz and C2-MeBz rings are twisted from the mean Bzm plane, the angles between the planes being 67.74 (4) (Bzm and N-nitroBz), 28.21 (5) (Bzm and C2-MeBz) and 64.77 (5)° (N-nitroBz and C2-MeBz), *i.e.* more twisted than in compound (II). The NO_2_ group is almost in the plane of the N-nitroBz ring [C14—C13—N13—O13*B* = 6.9 (2)°].

Mol­ecules of (III) are self-assembled in pairs through C11—H11⋯N3^i^ inter­actions in the form of an 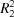
(12) ring (Fig. 5 ▸ *b*). Infinite tapes propagating along the *a*-axis direction are developed by C12—H12⋯N3^ii^ soft hydrogen bonds, forming an 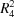
(10) ring motif (Fig. 5 ▸ *c*). Finally, the two- and three-dimensional structures are arranged through C14—H14⋯*Cg*2^iii^ and N13—O13*A*⋯*Cg*3^iv^ dispersive inter­actions [O13*A*⋯*Cg*3^iv^ = 3.254 (2) Å and N13⋯O13*A*⋯*Cg*3 = 94.12 (11)°; *Cg*2 is the centroid of the C4–C9 ring and *Cg*3 is the centroid of the C10–C15 ring; symmetry codes: (iii) −*x*, −*y* + 1, −*z*; (iv) −*x* + 1, −*y* + 1, −*z* + 2] (Fig. 5 ▸ *d*). The geometrical parameters and symmetry codes of the hydrogen bonding for compound (III) are listed in Table 6 ▸.

### Calculated mol­ecular structures of compounds (I)–(III)   

*Ab initio* theoretical density functional theory (DFT) calculations at the B3LYP/6-31G(d,p) level of theory were performed to support the experimental findings. The calculated geometric parameters are in agreement with the experimental ones. In general, the differences between the geometrical parameters in the experimental and optimized geometries are in most cases 0.01 Å (bond lengths) and 0.5° (bond angles), but large differences are observed for torsion angles that might be attributed to the gas-phase calculations without considering the crystal-packing forces. Additionally, the greater differences in favour of the N-nitroBz ring might be attributed to the presence of the nitro group, which is involved in inter­molecular inter­actions.

The nitro group retrieves electronic density from the benzene ring, so the C*sp* ^2^—H hydrogens bear a significant positive charge, particularly H12 and H14, which are both in *ortho* positions with respect to the nitro group. The calculated MKS charges are listed in Table 7 ▸. These H atoms lead to the formation of the hydrogen-bonding network in compound (I). The *n*–π* donor–acceptor inter­actions ONO⋯C*sp* ^2^ and N_py_⋯NO_2_ observed in (I) and (II), respectively, are charge assisted. In both mol­ecules, the N atom of the nitro group bears the most positive charge, followed by the C atom of the NCN fragment in the heterocyclic ring. In contrast, the pyridine-like N atom (N_py_) of the heterocycle bears the most negative charge, followed by the O atoms of the nitro group. The C2-Ph substitution in compound (II) has the effect of increasing the absolute value of the charges in the NCN fragment with the concomitant diminution of the dipolar moment [2.32 Debye in (I) to 1.84 Debye in (II)].

The theoretical energy profiles of compounds (I)–(III) were also calculated to estimate the energy involved in the inter­conversion between the N-nitroBz and C2-Ph rotamers. The experimental and theoretical torsion-angle values are listed in Table 8 ▸. The calculated most-stable rotamer of compound (I) is similar to that adopted by mol­ecule (I*A*) in the crystal lattice, where the N-nitroBz ring is twisted from the mean Bz plane, with a C8—N1—C10—C11 torsion angle of −39.43 (calculated) *versus* −32.47° (experimental). The maximum energy values of 3.27 or 2.10 kcal mol^−1^ were found when the N-nitroBz ring is coplanar or perpendicular to the benzimid­azole heterocycle, respectively. Because of symmetry reasons and the small cost in energy, the calculated rotamer with a C8—N1—C10—C11 torsion angle of 39.43° is equally probable (see Fig. S1 in the supporting information). The rotational barriers of compounds (I)–(III) are listed in Table S1 in the supporting information.

**Figure 6:**
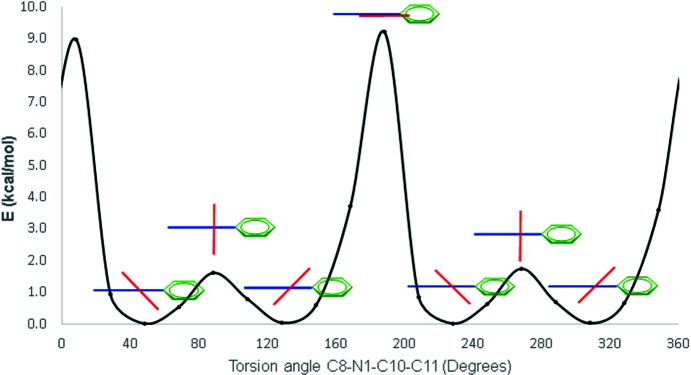
Theoretical rotation profile of the C8—N1—C10—C11 torsion angle in compound (II). The Bzm heterocycle in shown in blue, the N-nitroBz ring in red and the C2-Ph ring in green.

In the case of compound (II), the calculated C8—N1—C10—C11 and N1—C2—C16—C17 torsion angles of 58.60 and 33.73°, respectively, are in close correspondence with the mean absolute value of the four mol­ecules (II*A*)–(II*D*) found in the asymmetric unit (60.7±2.1 and 30.3±1.4°, respectively). The N1—C2—C16—C17 torsion angle was fixed at 33.73° to calculate the rotational barrier of the N-nitroBz ring. A maximum peak of energy 8.95 kcal mol^−1^ was found when the N-nitroBz ring is in the same plane as the Bz heterocycle and of only 1.60 kcal mol^−1^ when perpendicular (Fig. 6 ▸). Thus, the steric effect of the C2-Ph ring increases by 2.7-fold the energy required to rotate the N-nitroBz ring. The rotational barrier of the C2-Ph ring was calculated by fixing the C8—N1—C10—C11 torsion angle at 58.60°. Two energy maxima were found when the C2-Ph ring is perpendicular to or coplanar with the benzimidazole plane, with values of 3.60 and 0.99 kcal mol^−1^, respectively (see Fig. S2 in the supporting information). Therefore, the rotational barrier of the C2-Ph ring is just 40% of the energy required to rotate the N-nitroBz ring.

The calculated C8—N1—C10—C11 torsion angle of 58.72° contrasts with the experimental value of 72.23 (19)° for compound (III). This difference could be explained because of C11—H11⋯N3 hydrogen bonding to form the already described self-paired structure (*vide supra*). However, the energy profile and the maximum peak of energy 9.20 kcal mol^−1^ were found to be very similar to those exhibited by compound (II), in agreement with a negligible steric effect from the methyl group (see Figs. S3 and S4 in the supporting information).

**Figure 7:**
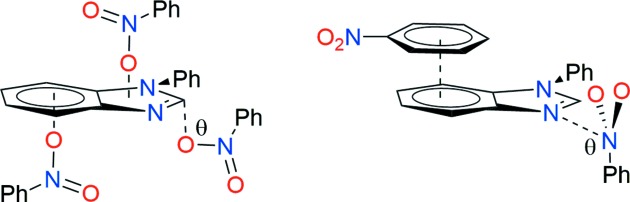
Pictorial representations of several inter­actions of the nitro group in the supra­molecular architecture of compounds (I)–(III), showing nitro–π* (left), and π–π* and N⋯NO_2_ (right). The θ angles are around 90°

In summary, compounds (I)–(III) are nonplanar mol­ecules having two strong hydrogen-bonding acceptors groups, *i.e.* an electron-withdrawing nitro group at one end and an electron-donating amino group at the other end. However, the structures lack strong hydrogen-bond donors. Thus, the crystal networks are developed by dispersive soft inter­actions, with the participation of the nitro group, namely C*sp* ^2^—H⋯ONO, 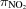
–π and *n*–π* (*n* = O and N_py_; π* = C*sp* ^2^ and 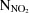) inter­­actions (Fig. 7 ▸).

Theoretical calculations confirmed that the presence of the C2-Bz ring increases the rotational barrier of the N-nitroBz ring, thus fewer conformers are expected. However, compound (II) is a high *Z*′-structure where equi-energetic conformers co-exist in the crystal network. Calculations also supported the fact that orthogonal ONO⋯C*sp* ^2^ and N_py_⋯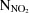 inter­actions are assisted by electrostatic attraction. The study of these mol­ecules bearing the benzimidazole pharmacophore and nitro­arenes would address the issues related to the steric and geometrical preferences for the occurrence of mol­ecular aggregation through nitro-group inter­actions with important pharmacological protein targets and the development of new materials. In this regard, a family of nitro­arene–benzimidazole compounds are under investigation as COX inhibitors by our research group.

## Supplementary Material

Crystal structure: contains datablock(s) I, II, III, global. DOI: 10.1107/S2053229618003406/uk3146sup1.cif

Structure factors: contains datablock(s) I. DOI: 10.1107/S2053229618003406/uk3146Isup2.hkl

Structure factors: contains datablock(s) II. DOI: 10.1107/S2053229618003406/uk3146IIsup3.hkl

Structure factors: contains datablock(s) III. DOI: 10.1107/S2053229618003406/uk3146IIIsup4.hkl

Theoretical rotation profiles and calculated rotation barriers. DOI: 10.1107/S2053229618003406/uk3146sup5.pdf

CCDC references: 1826170, 1565535, 1565536

## Figures and Tables

**Table 1:** Experimental details

	(I)	(II)	(III)
Crystal data			
Chemical formula	C_13_H_9_N_3_O_2_	C_19_H_13_N_3_O_2_	C_20_H_15_N_3_O_2_
*M*_r_	239.23	315.32	329.35
Crystal system, space group	Monoclinic, *C*2/*c*	Triclinic, *P* 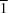	Triclinic, *P* 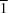
Temperature (K)	293	100	273
*a*, *b*, *c* (Å)	25.074 (3), 7.1422 (8), 24.283 (3)	10.2685 (7), 15.1411 (10), 19.4521 (14)	8.186 (4), 9.806 (4), 11.264 (5)
α, β, γ (°)	90, 96.599 (2), 90	91.886 (1), 95.725 (1), 90.118 (1)	112.825 (7), 98.468 (7), 94.276 (7)
*V* (Å^3^)	4319.9 (9)	3007.6 (4)	815.6 (6)
*Z*	16	8	2
Radiation type	Mo *K*α	Mo *K*α	Mo *K*α
μ (mm^−1^)	0.10	0.09	0.09
Crystal size (mm)	0.30 × 0.28 × 0.24	0.38 × 0.34 × 0.32	0.40 × 0.30 × 0.25

Data collection			
Diffractometer	Bruker APEXII area detector	Bruker APEXII area detector	Bruker APEXII area detector
No. of measured, independent and observed [*I* > 2σ(*I*)] reflections	19923, 3811, 3303	16980, 10449, 8103	9543, 3787, 3177
*R*_int_	0.047	0.029	0.022
(sin θ/λ)_max_ (Å^−1^)	0.595	0.595	0.666

Refinement			
*R*[*F* ^2^ > 2σ(*F* ^2^)], *wR*(*F* ^2^), *S*	0.072, 0.153, 1.20	0.054, 0.120, 1.05	0.050, 0.132, 1.04
No. of reflections	3811	10449	3787
No. of parameters	325	865	228
H-atom treatment	H-atom parameters constrained	H-atom parameters constrained	H-atom parameters constrained
Δρ_max_, Δρ_min_ (e Å^−3^)	0.24, −0.27	0.26, −0.22	0.19, −0.25

Computer programs: *APEX2* (Bruker, 2004 ▸), *SAINT* (Bruker, 2004 ▸), *SHELXS97* (Sheldrick, 2008 ▸), *SHELXL2014* (Sheldrick, 2015 ▸), *Mercury* (Macrae *et al.*, 2008 ▸), *SHELXL97* (Sheldrick, 2008 ▸) and *WinGX* (Farrugia, 2012 ▸).

**Table 2:** Hydrogen-bond geometry (Å, °) for (I)

*D*—H⋯*A*	*D*—H	H⋯*A*	*D*⋯*A*	*D*—H⋯*A*
C12—H12⋯O13*A* ^i^	0.93	2.45	3.350 (4)	164
C15—H15⋯O33*A* ^ii^	0.93	2.59	3.515 (4)	173
C24—H24⋯O33*B* ^iii^	0.93	2.55	3.375 (4)	149
C35—H35⋯O13*A* ^iv^	0.93	2.64	3.281 (4)	127

Symmetry codes: (i) 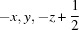; (ii) 
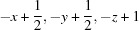
; (iii) 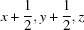; (iv) 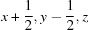.

**Table 3:** Experimental angles (°) between the planes of the Bzm, N-nitroPh and C2-Ph rings in mol­ecules *A*–*D* of compound (II)

Planes		Angles (°)					
1	2	(II*A*)	(II*B*)	Mean value	(II*C*)	(II*D*)	Mean value
				(II*A*) and (II*B*)			(II*C*) and (II*D*)
Bzm	N-nitroBz	54.20 (5)	54.40 (5)	54.30 (7)	60.47 (5)	58.02 (5)	59.25 (7)
Bzm	C2-Ph	29.05 (6)	29.10 (6)	29.08 (8)	31.22 (6)	32.74 (6)	26.98 (8)
N-nitroBz	C2-Ph	58.65 (5)	59.16 (5)	58.91 (7)	68.44 (6)	66.70 (5)	67.57 (8)

**Table 4:** Hydrogen-bond geometry (Å, °) for (II)

*D*—H⋯*A*	*D*—H	H⋯*A*	*D*⋯*A*	*D*—H⋯*A*
C6—H6⋯O13*A* ^i^	0.95	2.52	3.298 (3)	139
C7—H7⋯O33*B* ^ii^	0.95	2.54	3.357 (3)	145
C15—H15⋯O53*B* ^iii^	0.95	2.61	3.468 (3)	151
C26—H26⋯O33*B* ^iv^	0.95	2.52	3.302 (3)	140
C27—H27⋯O13*A* ^v^	0.95	2.53	3.355 (3)	145
C35—H35⋯O73*B* ^v^	0.95	2.64	3.527 (3)	156
C44—H44⋯O73*A* ^v^	0.95	2.66	3.309 (3)	126
C52—H52⋯O53*A* ^vi^	0.95	2.46	3.299 (3)	148
C54—H54⋯N3^vii^	0.95	2.47	3.412 (3)	171
C55—H55⋯O33*A* ^iv^	0.95	2.33	3.184 (3)	149
C64—H64⋯O53*A* ^viii^	0.95	2.51	3.227 (3)	132
C74—H74⋯N23^v^	0.95	2.50	3.437 (3)	167
C75—H75⋯O13*B* ^v^	0.95	2.34	3.217 (3)	153

Symmetry codes: (i) 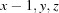; (ii) 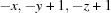; (iii) 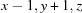; (iv) 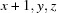; (v) 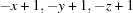; (vi) 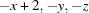; (vii) 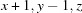; (viii) 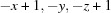.

**Table 5:** N⋯NO_2_ geometric parameters (Å, °) for (II)

C—N⋯N	N⋯N	C—N⋯N
C13—N13⋯N23^ix^	3.050 (2)	88.49 (19)
C33—N33⋯N3^*x*^	3.053 (2)	88.33 (19)
C53⋯N53⋯N63^viii^	3.102 (2)	90.58 (19)
C73⋯N73⋯N43^v^	3.134 (2)	91.59 (19)

Symmetry codes: (v) −*x* + 1, −*y* + 1, −*z* + 1; (viii) −*x* + 1, −*y*, −*z* + 1; (ix) *x*, *y*, *z*; (*x*) *x*, *y* − 1, *z*.

**Table 6:** Hydrogen-bond geometry (Å, °) for (III)

*D*—H⋯*A*	*D*—H	H⋯*A*	*D*⋯*A*	*D*—H⋯*A*
C11—H11⋯N3^i^	0.93	2.66	3.431 (2)	141
C12—H12⋯N3^ii^	0.93	2.47	3.348 (2)	157

Symmetry codes: (i) 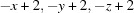; (ii) 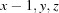.

**Table 7:** Selected MKS charges calculated at the B3LYP/6-31G(d,p) level of theory for compounds (I)–(III)

	MKS charge				MKS charge		
Atom	(I)	(II)	(III)	Atom	(I)	(II)	(III)
N1	−0.183	−0.324	−0.409	H4	0.183	0.172	0.164
C2	0.277	0.473	0.531	H6	0.142	0.133	0.133
N3	−0.597	−0.621	−0.541	H7	0.146	0.147	0.142
C10	0.185	0.303	0.359	H12	0.156	0.161	0.165
N13	0.661	0.648	0.659	H14	0.163	0.171	0.177
O13*A*	−0.394	−0.392	−0.396	H11	0.133	0.148	0.155
O13*B*	−0.394	−0.389	−0.393	H15	0.137	0.128	0.131

**Table 8:** Experimental and theoretically calculated torsion angles (°) in com­pounds (I)–(III)

	Mol­ecule	(I)	(II)	(III)
C8—N1—C10—C11	Calculated	−39.43	58.60	58.72
C8—N1—C10—C11	*A*	−32.47	−59.16	72.23 (19)
C28—N21—C30—C31	*B*	38.81	59.18	
C48—N41—C50—C51	*C*		63.54	
C68—N61—C70—C71	*D*		60.94	
N1—C2—C16—C17	Calculated		33.73	33.31
N1—C2—C16—C17	*A*		−29.19	29.5 (2)
N21—C22—C36—C37	*B*		29.69	
N41—C42—C56—C57	*C*		29.99	
N61—C62—C76—C77	*D*		32.26	
