# Supplementary material for: Involvement of conformational isomerism in the complexity of the crystal network of 1-(4-nitro­phen­yl)-1H-1,3-benzimidazole derivatives driven by C—H⋯A (A = NO2, Npy and π) and orthogonal Npy⋯NO2 and ONO⋯Csp 2 inter­actions
Source: Acta Crystallogr C Struct Chem. 2018 Mar 7;74(Pt 4):428–36. doi: 10.1107/S2053229618003406 (PMC5885323; doi:10.1107/S2053229618003406)
Supplement: Supplementary file 5 [file c-74-00428-sup5.pdf]

STRUCTURAL CHEMISTRY

Volume 74 (2018)

Supporting information for article:

Involvement of conformational isomerism in the complexity of the crystal network of 1-(4-nitrophenyl)-1*H*-1,3-benzimidazole derivatives driven by C—H···*A* (*A* = NO2, Npy and π) and orthogonal Npy···NO2 and ONO···C*sp*^{2} interactions

Mńica I. Garcá-Aranda, Carlos Z. Gomez-Castro, Efrén V. Garca-Báez, Yolanda Gómez y Gómez, José L. Castrejón-Flores and Itzia I. Padilla-Martínez

Supporting information

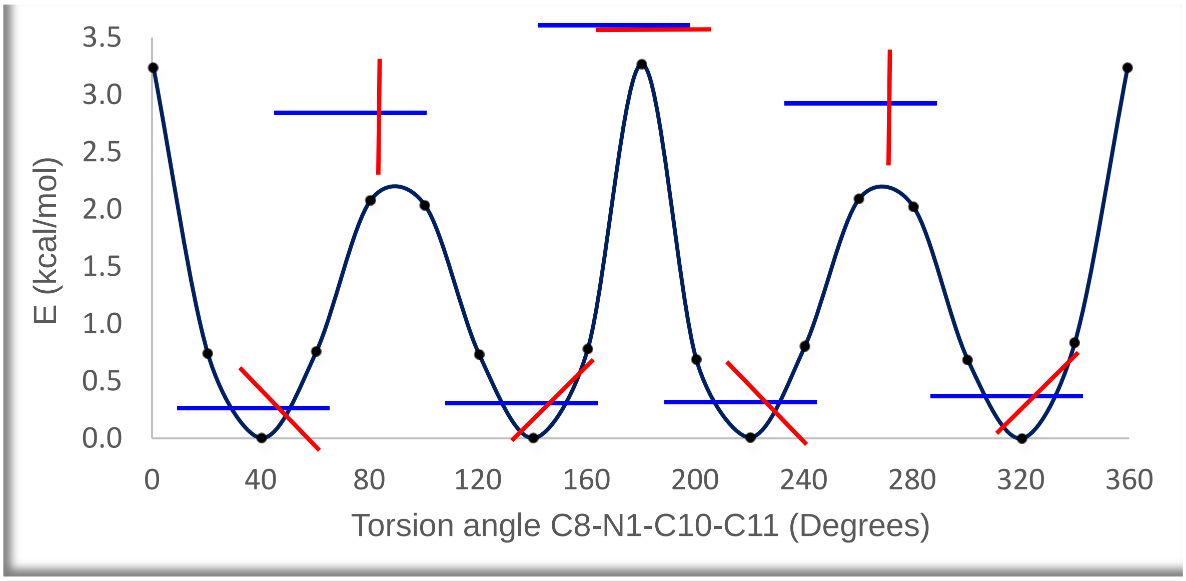

Figure S1 Theoretical rotation profile of the C8-N1-C10-C11 torsion angle in compound (I). Bz heterocycle in blue and *N*-nitroBz ring in red.

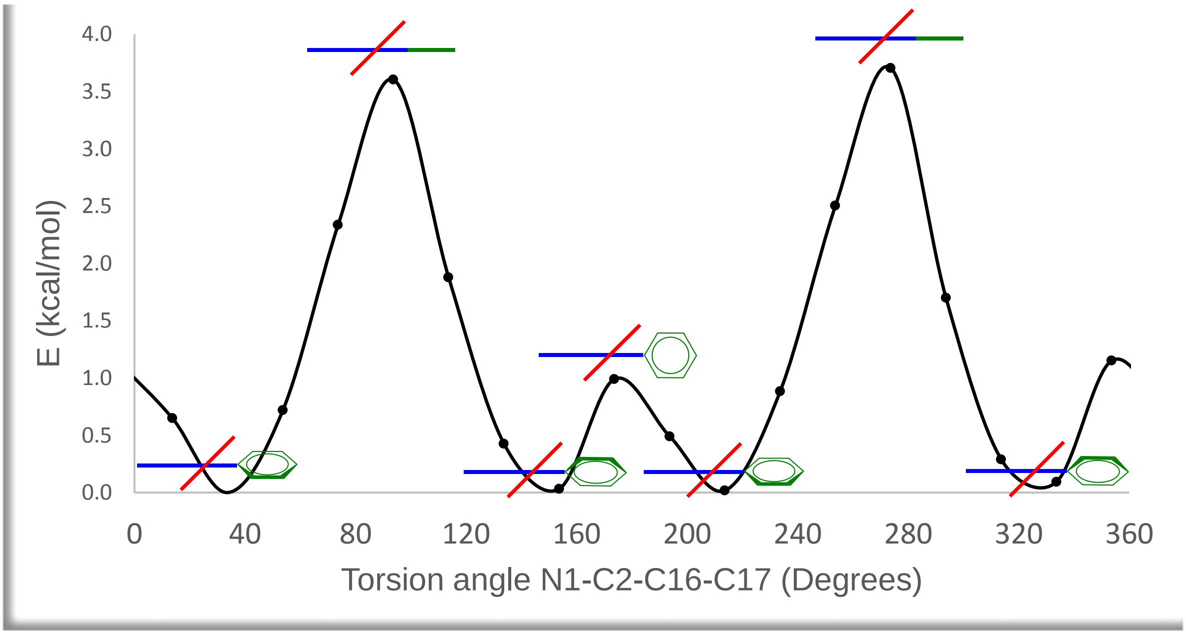

IMPORTANT: this document contains embedded data - to preserve data integrity, please ensure where possible that the IUCr Word tools (available from http://journals.iucr.org/services/docxtemplate/) are installed when editing this document.

Figure S2 Theoretical rotation profile of the N1-C2-C16-C17 torsion angle in compound (II). Bz heterocycle in blue, N-nitroBz ring in red and C-Ph ring in green.

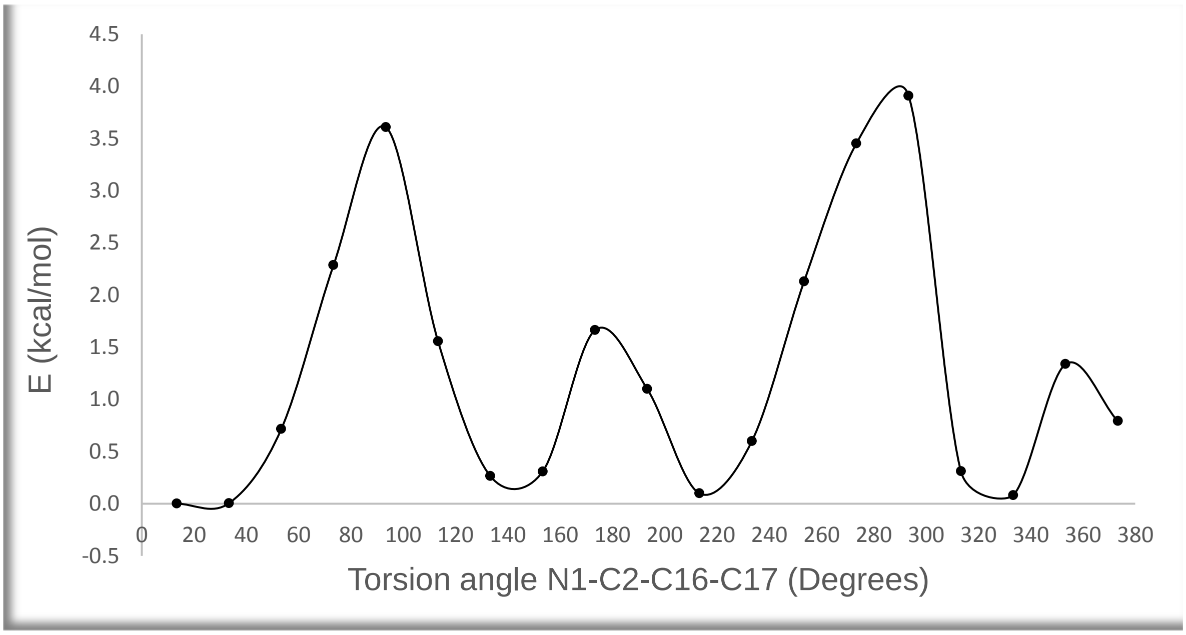

Figure S3 Theoretical rotation profile of the N1-C2-C16-C17 torsion angle in compound (III).

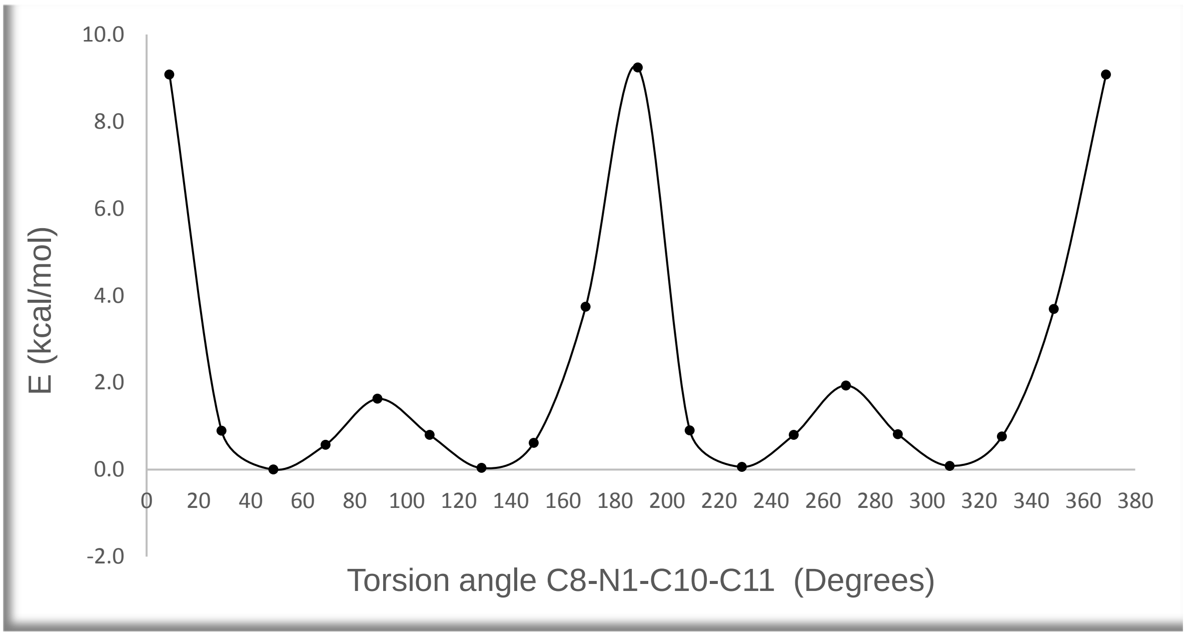

Figure S4 Theoretical rotation profile of the C8-N1-C10-C11 torsion angle in compound (III).

Table S1 Calculated rotation barriers (E_R in kcal mol^{-1}) at B3LYP/6-31G (*d*, *p*) level of theory in the rotation of C8-N1-C10-C11 (torsion angle 1), compounds (I)-(III), or N1-C2-C16-C17 (torsion angle 2), compounds (II) and (III)

Parameter	Compound

	(I)	(II)		(III)	
Fixed (°)		N1-C2-C16-C17 = 33.73	C8-N1-C10-C11 = 58.60	N1-C2-C16-C17 = 33.31	C8-N1-C10-C11 = 58.72
Rotating (°)	C8-N1-C10-C11	C8-N1-C10-C11	N1-C2-C16-C17	C8-N1-C10-C11	N1-C2-C16-C17
E_{R1} (kcal mol^{-1})	3.27	8.95	3.60	9.20	3.75
at (°)	180.57	8.57, 188.57	93.73, 273.73	188.81, 368.81.	93.35, 293.35
E_{R2} (kcal mol^{-1})	2.10	1.60	0.99	1.78	1.54
at (°)	90.57	88.57, 188.57	173.73, 353.73	88.81, 268.81	173.4, 353.4
